# A study of patient‐reported pain during bone marrow aspiration and biopsy using local anesthesia alone compared with local anesthesia with intravenous midazolam coadministration at a tertiary academic hospital in South Africa

**DOI:** 10.1002/hsr2.902

**Published:** 2022-10-31

**Authors:** Fatima Alzanad, Merga Feyaza, Zivanai C. Chapanduka

**Affiliations:** ^1^ Division of Hematological Pathology, Department of Pathology Stellenbosch University Faculty of Medicine and Health Sciences Cape Town South Africa; ^2^ National Health Laboratory Service Tygerberg Hospital Cape Town South Africa; ^3^ Division of Epidemiology, Department of Global Health Stellenbosch University Faculty of Medicine and Health Sciences Cape Town South Africa

**Keywords:** bone marrow aspiration and biopsy, intravenous midazolam, local anesthesia and benzodiazepine, patient reported pain

## Abstract

**Introduction:**

During the bone marrow aspiration and biopsy (BMAB) procedure, patients report pain of widely variable intensity. There is limited literature on the factors associated with the pain. The use of local anesthesia (LA) only is still widespread although it does not abolish the pain. Midazolam is the most commonly used benzodiazepine for conscious sedation. Our center introduced universal midazolam sedation unless there is a contraindication to its use, 4 years ago. This study assessed the impact of the universal use of intravenous midazolam for BMAB compared to use of LA only. The factors associated with the pain of BMAB, were analyzed.

**Methods:**

A retrospective cross‐sectional study was performed on adult patients who had a BMAB procedure from July 1, 2018 to March 30, 2019. A questionnaire incorporating a visual analog pain scale, was used for data collection.

**Results:**

A total of 182 BMAB procedures were included in the study. Pain was reported in all procedures performed under LA and only in 29.1% of procedures performed with midazolam. Age, sex, race, level of education, body mass index (BMI), indication and diagnosis had no influence on pain. Patients who had previous BMAB experienced less pain. Experience of operator had a significant effect on pain. Midazolam dose showed a negative correlation with pain.

**Conclusion:**

LA only is not enough to abolish pain of BMAB. Midazolam conscious sedation used with LA reduces pain to acceptable levels. Patients with previous experience of BMAB under midazolam premedication reported less pain. Furthermore, the experience of operator reduced the pain significantly.

## 1 INTRODUCTION

Bone marrow aspiration and biopsy (BMAB) is essential for the diagnosis, staging and monitoring of hematological disorders. BMAB also has an important role in the investigation of nonhematological condition such as fever of unknown origin and the diagnosis of infectious diseases, storage disorders and infiltration of the bone marrow by nonhematological malignancies.^1^ The procedure has evolved over time, with focus on easier and repeatable collection of bone marrow leading to full standardization of the procedure in the 1970s.^2^ BMAB is a painful procedure and the pain and anxiety associated with it, are variable and significant. The pain ranges from negligible mild pain, to distressing severe pain. There are limited studies focusing on the pain experienced during BMAB, the factors that influencing the pain and its management.^3^, ^4^

BMAB is usually performed as an outpatient procedure by trained clinicians of variable experience. The best site for the procedure is the iliac crest, posteriorly or anteriorly. Sternal puncture is performed in some situations, in which case only bone marrow aspiration is possible and sternal core biopsy is strictly prohibited. Bone marrow aspiration of the tibia can be performed in pediatric patients. Lignocaine (1% or 2%) is used for infiltrating the skin, subcutaneous tissue and periosteum. The standard technique should yield about 2 ml of bone marrow aspirate and a core biopsy about 20 mm length. A larger volume of aspirate is required for more investigations such as immunophenotyping, cytogenetics/molecular studies and microbiological cultures if needed.^5^, ^6^

The limited trials undertaken to reduce the pain associated with BMAB have not changed clinical practice and pain remains a heavy burden on hematology patients and the doctors performing the procedure. So far, there are insufficient evidence‐based guidelines to ameliorate pain resulting from BMAB.^7^ The management of the pain and anxiety becomes more critical because hematology patients often require several BMAB procedures.

Several factors influencing the pain experienced during BMAB have been studied. These include sex, age, body mass index (BMI), level of education, adequacy of information provided before the procedure, previous BMAB experience, level of expertise of the doctor performing the procedure (operator) and the duration and difficulty of the procedure. The results of the few studies focused on pain management during BMAB are contradictory.^3^, ^4^, ^7^

Several pharmacological and nonpharmacological methods have been proposed for pain reduction in patients undergoing BMAB.^3^ A local anesthetic (LA), usually lignocaine or a similar drug, is used to anesthetize the skin, subcutaneous tissue, and periosteum before the procedure.^5^ Conscious sedation is often used. Oral or intravenous benzodiazepines such as lorazepam, midazolam, and diazepam are the most commonly used sedatives for BMAB.^8^ In the United Kingdom, intravenous midazolam is routinely used for the BMAB procedure.^8^ Besides reducing anxiety and pain perception, benzodiazepines induce amnesia in most patients.^9^ Its rapid onset of action, short half‐life and the ease of availability of its antidote, flumazenil, make midazolam especially attractive in the setting of BMAB.

Chakupurakal et al.^8^ in a randomized controlled trial showed the superiority of the administration of midazolam with local anesthesia over Entonox in pain relief and reducing the recall of the procedure in patients undergoing BMAB.

It is the recall of any past pain which causes anxiety and may contribute to pain perception. In the study by Chakupurakal et al.,^8^ amnesia occurred in most patients who received midazolam sedation which reportedly made their BMAB experience acceptable. This made patients prefer midazolam sedation for subsequent BMAB.

Studies showed reduced pain intensity during BMAB performed with midazolam administration in conjunction with LA. Naumann et al.^10^ in a prospective study, compared the effect of relaxation (music and facultative instruction on how to relax), anxiolytic drug (midazolam), analgesic drug (piritramid), and placebo on pain reduction and anxiety in patients undergoing BMAB. Patients who received midazolam showed decreased pain intensity and increased threshold of pain compared to the other three groups in early and late evaluation, after 2 weeks.^10^

Glannoutosis et al.^11^ in a clinical trial, showed lower pain scores in patients who received midazolam in conjunction with local anesthesia (lignocaine) compared with patients who received local anesthesia only. The mean pain score by Visual Analog Score (VAS) was 1 in the midazolam group versus 3 in patients who received only LA. There was a lower level of apprehension toward having another BMAB in patients who received midazolam, however, the time required to perform the procedure was longer when using conscious sedation with an average of 30 min versus 21 min in patients receiving LA only.^11^ Stenstrup et al.^12^ evaluated the effect of using midazolam and LA for reducing anxiety and pain during BMAB and demonstrated that using midazolam and LA was comparable with the previous regimen of using opioids and lorazepam with the advantage of less side effects such as over sedation, which was a concern with using the combination of opioids and lorazepam.

This study aimed to assess the impact of the use of intravenous midazolam premedication for the BMAB procedure, using patient‐reported pain scoring. Furthermore, the factors associated with the attributes of the patient‐reported pain were determined.

## 2 MATERIALS AND METHODS

A retrospective cross‐sectional study was performed on adult patients (18 years old and more) who underwent the BMAB procedure at a day surgery operating theater (X‐block theater) over the 9‐month period from July 1, 2018 to March 30, 2019 at Tygerberg Hospital (TBH), Cape Town, South Africa. Cases in which the BMAB procedures were performed on the TBH wards, and cases where only bone marrow aspirates were obtained, with no core (trephine) biopsy, were excluded.

All the BMAB were performed on the iliac crest, in the lateral (fetal) position, and guided by a standard operation procedure (SOP) on which all operators were trained and assessed for competency.

Patients who had BMAB during the study period were interviewed during their normal follow‐up visits to the hematology clinic. Participants signed written informed consent to respond to the questions, to have their medical records viewed, and for their anonymized aggregated results to be published. Interviews were conducted over a 2‐month period by the principal investigator assisted by registered nurses who translated for those patients with limited English language ability. Patients were asked to answer an assigned questionnaire (Appendix S1).

A numerical rating scale (NRS) was used to categorize the pain experienced by the patient during the BMAB.^13^

It was explained to the patient as 0 no pain at all and 10 the worst imaginable pain ever. Pain scores were classified on an 11‐point scale from 0 to 10 as 0 equals no pain, (1‐3/10) mild pain, (4‐6/10) moderate, and (7‐10/10) severe pain. BMI calculations were categorized according to the World Health Organisation (WHO) report.^14^

Information regarding patient diagnosis and the doctor who performed the procedure were collected from patient medical records. Midazolam doses were extracted from medical records in operating theater.

IBM® Statistical Package for Social Sciences (SPSS® Inc) software for Microsoft Windows (version 16) was used for data analysis. Continuous data (age) was expressed as mean, median, and interquartile range (IQR). Categorical data were expressed as numbers and percentage. Evaluation of the association between variables and pain was performed by using the Mann–Whitney test and Chi‐square test. A *p*‐value ≤ 0.05 signifies statistical significance. Ethics approval for the study was granted by Stellenbosch University's Human Research Ethics Committee. Ethics number; S19/03/066.

## 3 RESULTS

One hundred and forty‐nine patients were enrolled on the study; 68 (45.6%) males and 81 (45.4%) females aged between 18 and 88 years. The summary of the basic patient characteristics are on Tables 1 and 2.

**Table 1:** Demographic characteristics of the study population, BMI, and indications of BMAB

Sex	*n* = 149
Male	68 (45.6%)
Female	81 (54.4%)
Male: female ratio	0.8:1
Age (years)	*n* = 149
Mean	49.2
Median	51.5
Interquartile range (years)	18–88
Race	*n* = 149
Black	52 (34.9%)
Colored	45 (30.2%)
White	29 (19.5%)
Indian	21 (14.1%)
Chinese	2 (1.4%)
Others	0 (0.0%)
Level of education	*n* = 149
No education	4 (2.7%)
Primary	32 (21.5%)
Secondary	80 (53.7%)
Tertiary	33 (22.1%)
BMI^a^	*n* = 182
Underweight	13 (7.1%)
Normal	66 (36.3%)
Overweight	65 (35.7%)
Obese	38 (20.9%)
Indication^a^	*n* = 182
Diagnostic	60 (32.9%)
Staging	48 (26.4%)
Follow‐up	74 (40.7%)

Abbreviations: BMAB, bone marrow aspiration and biopsy; BMI, body mass index.

^a^ The number of procedures is 182 and the number of patients is 149 because some patients had more than 1 BMAB procedure during the study period.

**Table 2:** Summary of adequacy of information, patient's previous experience, diagnosis, surgeon, midazolam administration, and dose of midazolam

Adequacy of information given to the patients	*n* = 182
Fully	147 (81.3%)
Partially	18 (9.9%)
None	17 (8.8%)
Previous BMAB patient's experience	n = 182
First time	123 (67.6%)
Repeat	59 (32.4%)
Diagnosis reported	n = 182
Acute leukemia	32 (17.6%)
MPN	22 (12.1%)
PMF	3 (1.6%)
NHL	57 (31.3%)
HL	16 (8.8%)
MDS	6 (3.3%)
AA	3 (1.6%
MM	20 (11.0%)
Plasmacytoma	4 (2.2%)
HLH	2 (1.1%)
Castleman disease	4 (2.2%)
Bicytopenia/pancytopenia	13 (7.2%)
Non‐hematological malignancy	1 (0.5%)
Surgeon	n = 182
First‐year registrars	77 (42.3%)
Second‐year registrars	34 (18.7%)
Fourth‐year registrars	9 (4.9%)
Fifth‐year registrars	62 (34.1%)
Medication	n = 182
Midazolam	155 (85.2%)
No midazolam	27 (14.8%)
Dose per kilogram	n = 108
Minimum	0.01
Maximum	0.121
Median	0.056
No dosage was recorded	47 (30.3%)

Abbreviations: AA, aplastic anemia; BMAB, bone marrow aspiration and biopsy; HL,  Hodgkin lymphoma; HLH, histiocytic lymphohistiocytosis; MDS, myelodysplastic syndrome; MM, multiple myeloma; MPN, myeloproliferative neoplasm; NHL, non‐Hodgkin lymphoma; PMF, primary myelofibrosis.

The majority of the procedures, 155 (85.2%) were performed after midazolam premedication with doses ranging from 0.01 mg/kg – 0.121 mg/kg. The rest 27 (14.8%) received lignocaine local anesthesia only.

Of the 155 procedures performed under midazolam premedication, pain was reported in 45 (29.1%) procedures, in contrast, all 27 (100%) procedures in the non‐premedicated group resulted in patient‐reported pain. This difference is statistically significant (*p* = <0.0001). Of the 45 procedures resulting in pain under midazolam premedication the majority resulted in mild pain 20 (44.4%), compared to only 8/27 (29.7%) in the non‐premedicated group (*p* = 0.0355). Moderate pain was experienced in 13/45 (28.9%) in the premedicated procedures compared to 9/27 (33.3%) in the non‐premedicated group (*p* = 0.0004). Severe pain was relatively less prevalent in the premedicated group 12/45 (26.7%) compared to 10/27 (37%) in the non‐premedicated group (*p* = 0.0003).

The mean midazolam dose in procedures resulting in pain was 0.043 mg/kg and in procedures which resulted in no pain was 0.061 mg/kg (*p* = <0.0001).

3.1

Showing that of the 45 patients experiencing pain in the midazolam group, pain scores ranged from 0 to 10 with a mean of (1.38), while in procedures performed under local anesthesia only pain scores ranged from 1 to 10 with a mean of 5.77 (*p* = <0.0001).

The presence of pain and the pain scores were not influenced by the patient's sex, age, race, level of education, BMI, quality of information given to the patient, indication for the BMAB and the diagnosis. See Tables 3 and 4.

**Table 3:** Patient demographic characteristics and pain score and grading

Factors	n	Mean	Min	Max	Mild, *n* = 28	Moderate, *n* = 22	Severe, *n* = 22	*p*‐value
Sex								
Male	34	1.77	1	10	17 (50%)	11 (32.4%)	6 (17.6%)	0.09
Female	38	2.29	1	10	11 (28.9%)	11 (28.9%)	16 (42.2%)	
Age								
Mean	72	NA	NA	NA	47.75	40.72	49.73	0.17
Race								
White	13	1.7	1	10	6 (46.1%)	4 (30.8%)	3 (23.1%)	0.85
Black	26	2.27	1	10	8 (30.8%)	10 (38.4%)	8 (30.8%)	
Indian	7	1.35	1	10	3 (42.9%)	1 (14.2%)	3 (42.9%)	
Chinese	0	0	1	0	0	0	0	
Colored	26	2.32	1	10	11 (42.3%)	7 (26.9%)	8 (30.8%)	
Level of education								
No education	2	1.25	2	3	2 (100%)	0	0	0.22
Primary	15	2.26	1	10	4 (26.7%)	3 (20%)	8 (53.3%)	
Secondary	41	2	1	10	18 (43.9%)	14 (34.1%)	9 (22%)	
Tertiary	14	1.97	1	10	4 (28.6%)	5 (35.7%)	5 (35.7%)	

**Table 4:** Grading of pain in relation to BMI, indication, and diagnosis

Factors	*n*	Mean	Min	Max	Mild, *n* = 28	Moderate, *n* = 22	Severe, *n* = 22	*p*‐value
BMI								
Underweight	5	1.92	3	8	2 (40%)	1 (20%)	2 (40%)	0.28
Normal	26	2.06	1	10	11 (42.3%)	4 (15.4%)	11 (42.3%)	
Overweight	24	2.03	1	10	7 (29.2%)	11 (45.8%)	6 (25%)	
Obese	17	2.05	2	10	8 (47.1%)	6 (35.3%)	3 (17.6%)	
Indication of BMAB								
Diagnostic	21	1.72	1	10	9 (42.9%)	7 (33.3%)	5 (23.8%)	0.85
Staging	26	3.06	1	10	8 (30.8%)	8 (30.8%)	10 (38.4%)	
Follow‐up	25	1.64	1	10	11 (44%)	7 (28%)	7 (28%)	
Diagnosis								
Acute leukemia	11	1.68	3	10	4 (36.4%)	5 (45.4%)	2 (18.2%)	0.51
MPN	10	2.18	1	7	3 (30%)	5 (50%)	2 (20%)	
PMF	1	1	3	3	1 (100%)	0 (0%)	0 (0%)	
NHL	27	2.7	1	10	9 (33.3%)	7 (25.9%)	11 (40.8%)	
HL	6	1.3	1	5	4 (66.7%)	2 (33.3%)	0	
MDS	0	0	0	0	0	0	0	
AA	1	1.3	4	4	0	1 (100%)	0	
MM	7	1.9	2	10	3 (42.9%)	1 (14.3%)	3 (42.8%)	
Plasmacytoma	1	0	0	0	1 (100%)	0	0	
HLH	2	4.5	1	8	1 (50%)	0	1 (50%)	
Castleman disease	3	3.5	3	7	1 (33.33%)	1 (33.33%)	1 (33.33%)	
Bicytopenia/pancytopenia	3	1.6	3	10	1 (33.3%)	0 (0%)	2 (66.7%)	

Abbreviations: AA, aplastic anemia; BMAB, bone marrow aspiration and biopsy; BMI, body mass index; HL,  Hodgkin lymphoma; HLH, histiocytic lymphohistiocytosis; MDS, myelodysplastic syndrome; MM, multiple myeloma; MPN, myeloproliferative neoplasm; NHL, non‐Hodgkin lymphoma; PMF, primary myelofibrosis.

Patients' previous BMAB experience had a significant influence on the presence of pain; pain was reported in 56 (45.5%) of procedures performed in patients who had no previous BMAB experience and in 16 (27.1%) of repeat procedures (*p* = 0.0076)

The mean pain score in the procedures performed on patients with a previous experience of BMAB was 1.25 which is lower than that of procedures performed on patients with no previous experience (3.41). Severe pain was reported in 17 (30.4%) procedures versus 5 (31.3%). This difference was statistically significant (*p* < 0.0063) (see Table 5).

**Table 5:** Grading of pain in relation to adequacy of information, previous patient's experience, and surgeon

Factors	*n*	Mean	Min	Max	Mild, *n* = 28	Moderate, *n* = 22	Severe, *n* = 22	*p*‐value
Adequacy of information								
Fully	57	1.93	0	10	22 (38.6%)	18 (31.6%)	17 (29.8%)	<0.076
Partially	8	2.33	0	10	3 (37.5%)	3 (37.5%)	2 (25%)	
None	7	2.58	0	9	3 (42.8%)	1 (14.3%)	3 (42.9%)	
Previous BMAB experience								
First time	56	3.41	0	10	21 (37.5%)	18 (32.1%)	17 (30.4%)	<0.0063
Repeat	16	1.25	0	10	7 (43.7%)	4 (25%)	5 (31.3%)	
Surgeon								
Junior registrars	52	2.47	0	10	25 (38.4%)	16 (30.8%)	16 (30.8%)	0.0113
Senior registrars	20	1.22	0	7	8 (40%)	6 (30%)	6 (30%)	

Abbreviation: BMAB, bone marrow aspiration and biopsy.

Ten procedures (62.5%) performed on patients with previous BMAB experience resulting in pain were performed with midazolam premedication. While only 6 (37.5%) were performed under LA. This difference is statistically significant (*p* = 0.0335).

Operator experience had a significant effect on pain (*p* = 0.0133). Pain was reported in 52 (46. 8%) of procedures performed by juniors while only 20 (28.2%) of procedures performed by seniors resulted in pain.

The lowest mean pain score 1.22 was in procedures performed by senior operator while the mean of pain scores in procedures performed by juniors was 2.47. The difference was statistically significant (*p* = 0.0113). (See Table 5).

## 4 DISCUSSION

Midazolam is the benzodiazepine most commonly used for sedation in medical procedures, especially for procedures outside the operating room. Its anxiolytic, sedative, and amnesia effects are of rapid onset and short duration, all of which are desirable attributes in the day‐surgery or bedside procedure settings.^15^ The majority of the procedures were performed under midazolam premedication and a minority received local anesthesia only. Patients with a contraindication to midazolam use received local anesthesia only. The contraindications included, patients with chronic heart failure, acute kidney injury, decreased lung function, and recreational drug abuse.

Our study shows that using local anesthesia only is not enough to abolish the pain of BMAB. All of the procedures performed with local anesthesia only resulted in pain. In sharp contrast, only 29.1% of procedures performed with midazolam sedation resulted in pain. Furthermore, the mean pain score was considerably lower in the midazolam premedication group compared with those who received local anesthesia only.

In a prospective interventional study, Naumann et al.^10^ showed reduced pain intensity and increased tolerance of pain when midazolam was used in patients undergoing BMAB. Giannoutosis et al.^11^ showed slightly lower pain scores (1 vs. 3) and lower levels of fear toward having a subsequent bone marrow examination in patients who received midazolam compared with patients who received local anesthesia only. Chakupurakal et al.^8^ in a randomized controlled trial, showed that Midazolam combined with local anesthesia provided rapid, reversible sedation and better pain control than nitric oxide combined with local anesthesia.

Doses of intravenous midazolam given to the patients ranged from 0.01 to 0.1 mg/kg. According to South African Society of Anaesthesiologists guideline for the safe use of procedural sedation and analgesia for diagnostic and therapeutic procedures in adults 2020–2025, the recommended IV midazolam dose is 0.05–0.1 mg/kg to a maximum bolus of 2 mg.^16^ In our study of the 182 procedures, doses were available for only 108 procedures, others were not available due to lost or misplaced theater records. There is a statistically significant negative correlation of dose of midazolam and the presence and severity of pain.

We found that variation in age had no statistically significant impact on the pain scores among the study subjects. This finding is similar to what was reported in previous studies. Degen et al.,^17^ in a prospective survey found that age had no impact on the pain reported in BMAB procedures. Contrary to our findings and the foregoing studies, Talamo et al.,^18^ in a prospective study showed a statistically significant decrease in pain with an increase in patient age, for patients undergoing BMAB.

There was no statistically significant difference in the pain reported by male patients compared with female patients. Our findings mirror those of Degen et al.^17^ who found no significant sex difference in pain perception in their cohort. The findings of Talamo et al.^18^ on the other hand, are contrary to our findings, they found that females reported a higher level of pain than males.

This study showed no difference in reported pain between patients regardless of the level of education. Furthermore, the reported degree of understanding of the BMAB had no influence on the pain experienced by the patient.

The level of education may be an important factor in the patient's ability to understand the explanation of the procedure. Wynia et al.^19^ found that limited patient literacy affects communication with patients regarding patient health care in general. In turn, patient understanding of the procedure may have an influence on pain perception and reporting. Gendron et al.^20^ showed a significant reduction in pain scores in well‐informed patients. Degen et al.^17^ showed that the patients who were less informed about key aspects of the BMAB procedure were more likely to experience pain than those who understood what the procedure entails.

This study comprises all the officially recognized ethnic groups in South Africa; Colored(mixed race), White, Indian and Asian. There was no difference in the reported pain after analysis by ethnic group. This is similar to the findings of Talamo et al.^18^  Studies have shown that there may be significant differences among ethnic groups with regard to pain perception.^21^ In view of South Africa's ethnic diversity, it was necessary to interrogate the role of ethnicity in the pain reported in this study.

Our study showed that BMI had no effect on the pain reported by patients.

The literature shows conflicting results regarding the association of BMI and pain during BMAB. Degen et al.^17^ and Talamo et al.^18^ found no difference in the pain experienced by patients of various BMI. Vanhelleputte et al.^22^ on the other hand, found that increased BMI was associated with increased pain during BMA. Admittedly, the medications and clinical circumstances of the procedures in the different studies may be significantly different.

Procedural difficulty is anticipated in BMAB performed on obese patients. In turn, procedural difficulty and prolongation of the time taken for the BMAB may increase the pain experienced by the obese patient.^5^

Despite the wide variety of indications for the BMAB procedure, there was no difference in patient‐reported pain regardless of whether the BMAB was performed as a diagnostic, follow‐up (or repeat), or staging procedure.

Patients who had a previous BMAB procedure reported less pain than BMAB naïve‐patients. This finding conflicts with some of the findings below. We did not match the individual patient's first and second experiences but rather, aggregated the reported pain. Following up individual patient experience on repeat BMAB may have given further insights into this finding.

Previous studies showed increased pain in patients with past BMAB experience due to increased anxiety or recall of a painful previous marrow.^17^ Other studies showed no association between the previous patient experience and pain. Vanhelleputte et al. showed no influence of previous experience on patient‐reported pain in a study performed on patients undergoing bone marrow aspiration only.^22^ Kuivalainen et al.^23^ demonstrated no difference, however, patients undergoing their first BMAB had lower pain scores during the injection of LA which is most likely due to administration of pre‐medication (oral diazepam or intramuscular alfentanil) according to patient request.

This study found no statistically significant correlation between the patient's diagnosis and pain, which is consistent with the findings of Gendron et al.^20^ Bone pain is part of the disease process in almost all multiple myeloma, in some acute leukemia and in other hematological conditions.^24^, ^25^ We, therefore, aimed to investigate the role of the underlying disease in the pain experience by our participants.

We showed a significant decrease in pain with increase in the experience of the operator. Previous studies were inconsistent. Degen et al.^17^ showed no effect of operator experience on patient reported pain. Kuball et al.^26^ in a prospective study showed reduction of duration of procedure with increased experience of the operator which influence pain.

## 5 LIMITATIONS

The questionnaire was applied at variable periods after the BMAB procedure with a minimum 2‐week gap. This makes it difficult to assess the role of patient memory in general and the role of the midazolam‐induced amnesia.

## 6 CONCLUSION

This study showed that local anesthesia is not enough to abolish the pain of BMAB and that midazolam is effective in pain control. Higher doses of midazolam were associated with lower pain scores. Patients who had previous BMAB experienced significantly less pain. Furthermore, the experience of the operator had a significant influence on pain. Age, sex, race, education, BMI, indication of BMAB, and diagnosis had no significant influence on the pain experienced. The universal use of safe and adequate midazolam premedication for BMAB is strongly recommended.

## AUTHOR CONTRIBUTIONS

**Fatima Alzanad**: Conceptualization; data curation; formal analysis; investigation; methodology; project administration; writing – original draft; writing – review & editing. **Merga Feyaza**: Formal analysis; methodology; writing – review & editing. **Zivanai Cuthbert Chapanduka**: Conceptualization; formal analysis; investigation; methodology; resources; supervision; validation; writing – review & editing.

## CONFLICTS OF INTEREST

The authors declare no conflicts of interest.

## TRANSPARENCY STATEMENT

The lead author Zivanai Cuthbert Chapanduka affirms that this manuscript is an honest, accurate, and transparent account of the study being reported; that no important aspects of the study have been omitted; and that any discrepancies from the study as planned (and, if relevant, registered) have been explained.

## Supporting information

Supporting information.

Supporting information.

Supporting information.

Supporting information.

Supporting information.

## Data Availability

All data associated with this study are available from the corresponding author via Email: zivanai@sun.ac.za.

## References

[1] Kaur M , Singh Rana AP , Kapoor S , Puri A . Diagnostic value of bone marrow aspiration and biopsy in routine haematology practice. J Clin Diagn Res. 2014;8(8):FC13‐FC16. 10.7860/JCDR/2014/9823.4760 PMC4190721 25302200

[2] Zahid M . Methods of reducing pain during bone marrow aspiration and biopsy. Ann Palliat Med. 2015;4(4):184‐193. 26541397 10.3978/j.issn.2224-5820.2015.09.02

[3] Hjortholm N , Jaddini E , Hałaburda K , Snarski E . Strategies of pain reduction during the bone marrow biopsy. Ann Hematol. 2013;92(2):145‐149. 23224244 10.1007/s00277-012-1641-9 PMC3542425

[4] Watmough S , Flynn M . A review of pain management interventions in bone marrow biopsy. J Clin Nurs. 2011;20(5‐6):615‐623. 21320190 10.1111/j.1365-2702.2010.03485.x

[5] Bain B , Bates I , Laffan M . Dacie and Lewis‐Practical haematology 11th Edition. 11 ed. Churchill Livingstone; 2011.

[6] Trejo‐Ayala RA , Luna‐Pérez M , Gutiérrez‐Romero M , Collazo‐Jaloma J , Cedillo‐Pérez MC , Ramos‐Penafiel CO . Bone marrow aspiration and biopsy. Technique and considerations. Rev Méd Hospital Gen México. 2015;78(4):196‐201.

[7] Bain BJ . Bone marrow aspiration. J Clin Pathol. 2001;54(9):657‐663. 11533068 10.1136/jcp.54.9.657 PMC1731527

[8] Chakupurakal G , Delgado J , Nikolousis E , et al. Midazolam in conjunction with local anaesthesia is superior to entonox in providing pain relief during bone marrow aspirate and trephine biopsy. J Clin Pathol. 2008;61(9):1051‐1054. 18755727 10.1136/jcp.2008.058180

[9] Mainwaring CJ , Wong C , Lush RJ , Smith JG , Singer CR . The role of midazolam‐induced sedation in bone marrow aspiration/trephine biopsies. Clin Lab Haematol. 1996;18(4):285‐288. 9054704

[10] Naumann R , Schneider E , Einsle F , et al. Midazolam: significant pain reduction in patients undergoing bone marrow puncture‐a clinical trial. Blood. 2004;104(11):90.

[11] Giannoutsos I , Grech H , Maboreke T , Morgenstern G . Performing bone marrow biopsies with or without sedation: a comparison. Clin Lab Haematol. 2004;26(3):201‐204. 15163318 10.1111/j.1365-2257.2004.00608.x

[12] Stenstrup E , Skendzel S , Warlick E , Parran L . Reducing the risk of sedation for adult bone marrow biopsy patients: establishing a new protocol to support pain control and anxiolysis. Biol Blood Marrow Transplant. 2017;23(3):S452.

[13] Breivik H , Borchgrevink PC , Allen SM , et al. Assessment of pain. Br J Anaesth. 2008;101(1):17‐24. 18487245 10.1093/bja/aen103

[14] WHO Consultation on Obesity (‎1999: Geneva, Switzerland)‎ & World Health Organization. Obesity: preventing and managing the global epidemic: report of WHO consultation. World Health Organization. 2000. Accessed on March, 20, 2022. https://apps.who.int/iris/handle/10665/42330 11234459

[15] Jo YY , Kwak HJ . Sedation strategies for procedures outside the operating room. Yonsei Med J. 2019;60(6):491‐499. 31124331 10.3349/ymj.2019.60.6.491 PMC6536395

[16] Guidelines for the safe use of procedural sedation and analgesia for diagnostic and therapeutic procedures in adults: 2020–2025. Southern Afr J Anaesth Analgesia. 2020;26(2):S1‐S75.

[17] Degen C , Christen S , Rovo A , Gratwohl A . Bone marrow examination: a prospective survey on factors associated with pain. Ann Hematol. 2010;89(6):619‐624. 20333524 10.1007/s00277-010-0934-0

[18] Talamo G , Liao J , Bayerl MG , Claxton DF , Zangari M . Oral administration of analgesia and anxiolysis for pain associated with bone marrow biopsy. Supp Care Cancer. 2010;18(3):301‐305. 10.1007/s00520-009-0652-0 19455356

[19] Wynia MK , Osborn CY . Health literacy and communication quality in health care organizations. J Health Commun. 2010;15(S2):102‐115. 10.1080/10810730.2010.499981 PMC3086818 20845197

[20] Gendron N , Zia Chahabi S , Poenou G , et al. Pain assessment and factors influencing pain during bone marrow aspiration: a prospective study. PLoS One. 2019;14(8):e0221534. 31465426 10.1371/journal.pone.0221534 PMC6715342

[21] Edwards RR , Doleys DM , Fillingim RB , Lowery D . Ethnic differences in pain tolerance: clinical implications in a chronic pain population. Psychosom Med. 2001;63(2):316‐323. 11292281 10.1097/00006842-200103000-00018

[22] Vanhelleputte P , Nijs K , Delforge M , Evers G , Vanderschueren S . Pain during bone marrow aspiration: prevalence and prevention. J Pain Symptom Manage. 2003;26(3):860‐866. 12967736 10.1016/s0885-3924(03)00312-9

[23] Kuivalainen AM , Pitkäniemi J , Widenius T , Elonen E , Rosenberg P . Anxiety and pain during bone marrow aspiration and biopsy. Scand J Pain. 2012;3(2):92‐96. 29913774 10.1016/j.sjpain.2011.11.002

[24] Diaz‐delCastillo M , Andrews RE , Mandal A , Andersen TL , Chantry AD , Heegaard AM . Bone pain in multiple myeloma (BPMM)—a protocol for a prospective, longitudinal, observational study. Cancers. 2021;13(7):1596. 33808348 10.3390/cancers13071596 PMC8036720

[25] Morais SA , du Preez HE , Akhtar MR , Cross S , Isenberg DA . Musculoskeletal complications of haematological disease. Rheumatology. 2016;55(6):968‐981. 26443208 10.1093/rheumatology/kev360

[26] Kuball J , Schüz J , Gamm H , Weber M . Bone marrow punctures and pain. Acute Pain. 2004;6(1):9‐14.

