# Supplementary material for: A study of patient‐reported pain during bone marrow aspiration and biopsy using local anesthesia alone compared with local anesthesia with intravenous midazolam coadministration at a tertiary academic hospital in South Africa
Source: Health Sci Rep. 2022 Oct 31;5(6):e902. doi: 10.1002/hsr2.902 (PMC9621466; doi:10.1002/hsr2.902)
Supplement: Supplementary file 1 — Supporting information. [file HSR2-5-e902-s001.docx]

**8 Questionnaire and data capturing sheath**

1. Name: _____________________________________________________________________
2. Folder number: ______________________________________________________________
3. ST number (s): _______________________________________________________________
4. Gender

- Male
- Female

1. Age: _________ years and fraction thereof
2. Race

- Black or African
- Chinese
- Coloured
- Indian
- White
- Other

1. Weight

________in kilograms

1. Height

in centimetres

1. Body mass index (BMI) = Weight/ height squared
2. Level of education

- Primary
- Secondary
- Tertiary

1. How many bone marrow procedures done in the past?

___________ numerical

1. What is the haematological diagnosis(es) If known?

______________________________________________

______________________________________________

______________________________________________

1. What is the reason/indication of the bone marrow procedure?

______________________________________________

______________________________________________

______________________________________________

1. Did the patient receive premedication?

- Yes
- No

1. What type of premedication in addition to local anaesthetic?

- Midazolam (intravenous)
- Lorazepam (oral)

**Pain Grading Questionnaire**

To assess the pain (if any) that you may have felt during the bone marrow specimen collection procedure, we have graded the pain on a scale of 0 – 10 in which 0 means that you felt no pain at all and 10 is the worst pain you can imagine.

- 0 -No pain felt
- 1
- 2
- 3
- 4
- 5
- 6
- 7
- 8
- 9
- 10 – severest / worst possible pain

**Notes**

Please enter nay relevant notes about the patient, the procedure etc.

________________________________________________________________________________________________________________________________________________________________________________________________________________________________________________________________________________________________________________________________________________________________________________________________________________________________________________________________________________________________________________________________________________________________________________________________________________________________________________________________________________
