# Supplementary material for: A study of patient‐reported pain during bone marrow aspiration and biopsy using local anesthesia alone compared with local anesthesia with intravenous midazolam coadministration at a tertiary academic hospital in South Africa
Source: Health Sci Rep. 2022 Oct 31;5(6):e902. doi: 10.1002/hsr2.902 (PMC9621466; doi:10.1002/hsr2.902)
Supplement: Supplementary file 2 — Supporting information. [file HSR2-5-e902-s005.pdf]

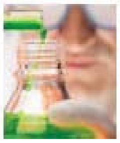

STANDARD OPERATING PROCEDURE

Title: How to perform a bone marrow aspirate and trephine biopsy

Document number: HAE1188

Version number: 2

(Changes from previous version highlighted)

Written by: Dr. C. Griesel

Checked by: Dr. Z.C Chapanduka

Approved by: Dr Z.C Chapanduka

Active date: 13-08-2018

Date of next review	*Date reviewed*	*Reviewed by*	*Action*
13-08-2019			

Date withdrawn: ……………..

In the event of a dispute concerning this document, the electronic version stored on Q-Pulse will be deemed to be the correct version

National Health Laboratory Service- All rights reserved

OBJECTIVE

To provide Haematological and Clinical Pathology Registrars with clear guidance on the process of obtaining a bone marrow aspirate and biopsy.

INTRODUCTION AND BACKGROUND

Clinicians request bone marrow aspiration and biopsy (BMAB) for diagnosis, staging and monitor of lympho-haematological, metastatic, infectious and granulomatous diseases. Contra-indications include a significant coagulopathy, Haemophilia or infection at the site of incision. Patients with respiratory problems or other serious co-morbidities, patients who cannot be placed in the best position for the procedure to be successfully completed and patients deemed too ill and/or unstable should be deferred until it is safe to proceed.

RESPONSIBILITY

Clinical Pathology Registrars rotating in Haematology
Haematological Pathology Registrars
Technologists
Consultants.

PREPARATION

1. Booking of the procedure should be done according to the booking SOP (HAE0882).
2. Clinical haematology, other clinical disciplines as well as peripheral clinics can make a booking, after consultation with Clinical Haematology.
3. The adult bone marrow procedure is performed by a Registrar or Consultant Haematopathologist. The Paediatric bone marrow procedure is performed by paediatricians on the ward.
4. At Tygerberg Hospital bone marrow procedures are performed in a fully equipped theatre in X-block daily between Monday and Friday. The occasional procedure may be done in other locations by special arrangement.
5. There is a dedicated nurse in attendance at all times.
6. The attending physician is responsible for explaining the procedure to the patient and obtaining informed consent.
7. The attending physician should prescribe the Midazolam that will be administered for conscious sedation.
8. The ward nursing staff is involved in the post-procedural care of the incision site and must observe the patient's vital signs, for bleeding and general stability.

EQUIPMENT AND MATERIALS

1. Special Bone Marrow sterile pack containing paper handtowels, surgical drapes, forceps, artery clamps, scalpel and blade, cotton wool swabs and gauze swabs
2. Disposable aspirate and trephine needle.
3. Sterile gloves, protective eye goggles and plastic apron.
4. Local anaesthetic: 1% or 2% lignocaine
5. Syringes 20ml, 10ml and 5ml
6. Needles: 21G or spinal needle for obese patients.
7. Skin cleaning agent
8. Specimen tubes: EDTA, Citrate, Heparin and Plain
9. Fixative for the trephine biopsy – formalin based.
10. Microscope slides and slide-tray

In the event of a dispute concerning this document, the electronic version stored on Q-Pulse will be deemed to be the correct version

National Health Laboratory Service- All rights reserved

SAFETY PRECAUTIONS.

It is critical to ensure the safety of the patient, the staff and the surgeon.

The risks, including needle-stick transmission of disease, transmission of airborne infections and other infections transmitted through fluid-to-mucosa contact should be minimised by following universal protective measures including but not limited to:

Obtaining history of risk before performing the procedure, specifically, history of TB and/or MDR/XDR TB should be elicited and extra precautions taken.

Observance of sterile procedure

Protective eye wear,

Gowns and

Gloves

INFORMED PATIENT CONSENT:

It is the responsibility of the clinician referring the patient to ensure that written informed consent is obtained and appropriately filed.

It is the duty of the doctor performing the BMAB procedure to physically inspect and determine the adequacy of the consent form; the procedure should not be performed without signed informed consent.

PROCEDURE/METHOD

1. General measures

a. Ensure that the patient has a good free-flowing IV cannula kept open with a 200ml bag of normal saline.
b. Secure a baseline Oxygen saturation (SaO2) by pulse-oximetry >/= 90% on room air.
c. If baseline SaO2 </=84% on room air, inform a Consultant before proceeding.
d. If SaO2 >84% <90% give oxygen by mask to correct SaO2 to >90%
e. If SaO2 < 90% on oxygen inform a Consultant before proceeding.
f. Dilute Midazolam 5 mg to 10 ml with normal saline (2ml = 1mg Midazolam) and keep ready.
g. Ensure the availability of Flumazenil (1mg) before the use of Midazolam in case of need for reversal of the effects of Midazolam

2. Aspiration from the posterior iliac crest

a. Position the positioned in the lateral decubitus position, also known as the lumbar puncture or foetal position; both legs are flexed with the upper leg flexed higher than the lower leg.
b. Identify both the posterior and anterior superior iliac spine, determine the orientation of the iliac bone, the best accessible bony prominence and the amount of tissue overlying it.
c. In case of thick overlying tissue, a spinal needle must be used for infiltrating local anaesthesia and the longer biopsy needle must be used.
d. Give the IV Midazolam slowly until the effect first signs of effect on the patient can be seen, a maximum dose of 5mg is allowed. If higher doses are required Consultant permission must be obtained.
e. Ensure that SaO2 is >90% before proceeding.
f. Clean the biopsy site with an antiseptic scrub and apply drapes, exposing only the biopsy site.
g. Infiltrate the skin and subcutaneous tissue with a few ml of Lignocaine and use the rest of the Lignocaine for thorough infiltration of the periosteum. Use generous amounts of Lignocaine ensuring that intravenous injection does not happen.
h. Make a skin incision, maximum 4mm long with a surgical blade. Ideally a size 10 curved blade.

In the event of a dispute concerning this document, the electronic version stored on Q-Pulse will be deemed to be the correct version

National Health Laboratory Service- All rights reserved

i. A standard length Jamshidi type needle size 8G or 11G is used to obtain both the aspirate and trephine sample. An extra length needle will be required for obese patients.
j. The bone marrow aspiration needle, with a stylet in place, is inserted. Once the needle contacts the bone, it is advanced by rotating clockwise and counter clockwise slowly until the marrow cavity is entered. Usually, a "give" is felt when the marrow cavity is entered. However, this "give" may not be felt if the patient is severely osteoporotic or osteosclerotic, or in the presence of fibrosis.
k. Once needle is placed within the marrow cavity, remove the stylet and, using a 5ml or 10ml syringe, aspirate approximately 0.5-1ml of bone marrow. No more than 2 ml of aspirate should be obtained for slide-making, to avoid haemodilution.
l. Aspiration with the needle appropriately placed, often results in a sharp pain to the patient.
m. Quickly hand the aspirate to the technologist for slides to be made on the fresh aspirate before it clots.
n. If additional tests such as flow cytometry, cultures and cytogenetics are required, a clean 10ml or 20ml syringe should be used.
o. In case there is no technologists available or if excess aspirate is obtained, it may be placed in EDTA and slides made in the laboratory. Note that placing aspirate in EDTA introduces several distinct artefacts therefore the aim should be to make fresh slides without EDTA.
p. If the patient complains of clear "aspiration pain" but no aspirate is obtained, no further attempts to aspirate should be made as this is due to inspirable marrow such as in myelofibrosis, some acute leukaemias and metastatic malignancies.
q. Inability to obtain an aspirate a "dry tap" may be due failure to enter the marrow cavity. In that case replace the stylet and carefully advance the needle by a few mm, remove the stylet and aspirate again.
r. Attempted aspiration during careful retraction of the needle may yield some aspirate.
s. Bone marrow biopsy is usually done following aspiration.
t. If biopsy is not required a medicated sterile pressure dressing and manual pressure for 3 – 5 minutes should be applied to stop bleeding and minimise haematoma formation.

3. Trephine/core biopsy from the posterior iliac crest

a. The trephine biopsy is obtained using the same needle.
b. If the same puncture site is used, a longer biopsy must be obtained to counter the effect of aspiration induced hypocellularity.
c. If a biopsy is being performed for staging purposes, two puncture sites at least 1cm apart are required, alternatively, a bilateral biopsy may be obtained.
d. The needle is held with the palm and index finger, and the stylet is locked in place.
e. Using firm pressure, slowly rotate the needle in an alternating clockwise-counter clockwise motion and advance it through the cortical bone until the bone marrow cavity is reached.
f. Rotate the needle 20 times in a clockwise direction, followed by several clockwise-anticlockwise rotations until the biopsy needle feels loose.
g. Pull back approximately 2-3 mm, and advance the needle again slightly, at a different angle, to help break off the core biopsy.
h. Slowly pull the needle out while rotating in an alternating clockwise and counter clockwise motion and feeling the needle's movement carefully
i. Remove the specimen from the needle with the probe supplied by introducing the probe through the distal cutting end.
j. The core biopsy is used to make touch preparations prior to placing the specimen in fixative solution.
k. Gently rolling the biopsy on clean slides until it is dry is recommended but several touch/crush preparation-making techniques may be used.
l. Care should be taken not to distort the biopsy.
m. Place the specimen in the fixative solution for histology processing.

In the event of a dispute concerning this document, the electronic version stored on Q-Pulse will be deemed to be the correct version

National Health Laboratory Service- All rights reserved

n. A medicated sterile pressure dressing and manual pressure for 3 – 5 minutes should be applied to stop bleeding and minimise haematoma formation.

4. Processing of specimens

a. A trained, qualified and accredited technologist should process the bone marrow sample.
b. Careful labelling and documentation is critical.
c. The pathologist selects the slides for May Grunewald-Giemsa and Iron stains ensuring that enough unstained slides are kept in case of future need.
d. The trephine biopsy is recorded and taken to Anatomical Pathology in the shortest possible time.

ANALYSIS/INTERPRETATION OF RESULTS

a. The technologist ensures that the bone marrow slides are stained, quality checked, labelled and ready for pathologist examination within the shortest time to conform with Turn Around Times (TAT)
b. Timing and quality expectations, roles and responsibilities are defined and agreed upon.
c. The Registrar performs the preliminary examination and presents the findings to a Consultant for finalisation and authorisation.

REFERENCE RANGES AND EXPECTED RESULTS

Myelogram ranges for age and gender are provided.
Guidelines for the assessment of iron and reticulin stain are available.
For interpretation of special cytochemical and immunohistochemical stains refer to the applicable SOP.

PROCEDURE FOR ABNORMAL RESULTS

It is the responsibility of the Registrar and Consultant to communicate abnormal and/or urgent bone marrow results to the patient's treating doctors.

REFERENCES

1.Practical Haematology 9th Edition Dacie and Lewis pg101-114.

2. WHO Model Prescribing Information: Drugs Used in Anaesthesia

In the event of a dispute concerning this document, the electronic version stored on Q-Pulse will be deemed to be the correct version

National Health Laboratory Service- All rights reserved

Acknowledgement of Reading Form

Document number: ________________ **Version Number:** ________________

Title: ________________

My signature confirms that I have read and understood the content of this document and relevant kit insert (where applicable).

Name	Signature	Date

Note to the Quality Rep: - This form must be filed for 5 years to provide audit traceability.

In the event of a dispute concerning this document, the electronic version stored on Q-Pulse will be deemed to be the correct version

National Health Laboratory Service- All rights reserved
