# Supplementary material for: A study of patient‐reported pain during bone marrow aspiration and biopsy using local anesthesia alone compared with local anesthesia with intravenous midazolam coadministration at a tertiary academic hospital in South Africa
Source: Health Sci Rep. 2022 Oct 31;5(6):e902. doi: 10.1002/hsr2.902 (PMC9621466; doi:10.1002/hsr2.902)
Supplement: Supplementary file 3 — Supporting information. [file HSR2-5-e902-s004.docx]

PARTICIPANT INFORMATION LEAFLET AND CONSENT FORM

| **Title of Research Project:** | |
| --- | --- |
| **Study of patient-reported pain during Bone Marrow Aspiration and Biopsy using local anaesthesia with intravenous midazolam co-administration at Tygerberg Hospital in South Africa.** | |
| **DETAILS OF PRINCIPAL INVESTIGATOR (PI):** | |
| **Title, first name, surname: Dr Fatima Alzanad** | **Ethic reference number:**  **S19/03/066** |
| **Full postal address:** | **PI Contact number:** |

We would like to invite you to take part in a research project. Please take some time to read the information presented here, which will explain the details of this project. Please ask the study staff or doctor any questions about any part of this project that you do not fully understand. It is very important that you are completely satisfied that you clearly understand what this research entails and how you could be involved. Also, your participation is **entirely voluntary,** and you are free to decline to participate. In other words, you may choose to take part, or you may choose not to take part. Nothing bad will come of it if you say no: it will not affect you negatively in any way whatsoever. Refusal to participate will involve no penalty or loss of benefits or reduction in the level of care to which you are otherwise entitled to. You are also free to withdraw from the study at any point, even if you do agree to take part initially.

This study has been approved by the Health Research Ethics Committee at Stellenbosch University. The study will be conducted according to the ethical guidelines and principles of the international Declaration of Helsinki, the South African Guidelines for Good Clinical Practice (2006), the Medical Research Council (MRC) Ethical Guidelines for Research (2002), and the Department of Health Ethics in Health Research: Principles, Processes and Studies (2015).

## What is this research study all about?

This study is meant to find out if the medicines we currently use to perform bone marrow biopsies are good enough to ensure that the patient does not feel pain during the biopsy procedure.

We will therefore ask all those patients on whom we did bone marrow biopsies questions to find out if they felt pain during the procedure or not. In cases in which any pain was felt we want to know how much pain they experienced.

You had a bone marrow biopsy procedure done and we are therefore very much interested to know your experience.

## Why do we invite you to participate?

You are chosen to be part of the study because you had a bone marrow biopsy performed in the X block theatre. We therefore want to know of your experiences.

## What will your responsibilities be?

You will be asked to answer some questions, which will be on the same questionnaire for all patients taking part in this study. Your responsibility is to answer all the questions truthfully as far as your memory serves you. We are asking for not more that 5 – 10 minutes of your time.

## Will you benefit from taking part in this research?

There is no direct benefit to you as an individual. However, this study will help us to decide on the best way to ensure that patients who have bone marrow biopsies do not suffer any pain.

## Are there any risks involved in your taking part in this research?

This study poses no serious risk to you. However, because of the possibility that you may have experienced pain, remembering such pain has been shown to cause anxiety and unhappiness in some patients. If during the questioning, you feel upset or anxious, please inform our research team and know that you are free to stop the questioning. If you feel upset or anxious after the questioning, please inform the research team and we will refer you to Mr Wood who is trained to assist you.

## If you do not agree to take part, what alternatives do you have?

You can choose whether to be in this study or not. If you agree to take part in this study, you may withdraw at any time without any negative consequence. You may also refuse to answer any questions you don’t want to answer without suffering any negative consequences.

## Who will have access to your medical records?

Only Dr Fatima Alzanad (the principal researcher) and Dr Zivanai Chapanduka (her supervisor) will have access to the information.

The signed consent form and other documents containing identifiable data will be kept under lock in a secured location.

Any item that may be used to identify you will be removed and destroyed as soon as possible and access to research data will be based on an absolute need to know basis and only with the permission of Drs Alzanad and Chapanduka.

The results will be summarized and reported together as a group. Your name or any other information that may identify you will not be shared. When the findings of this study are published or presented at scientific conferences we will ensure that you identity cannot be revealed.

Will you be paid to take part in this study and are there any costs involved?

You will not be paid for answering the questions.

Is there anything else that you should know or do?

Please feel free to phone Dr Fatima Alzanad at (…..) or Dr Chapanduka on (……) if you have any further queries or encounter any problems.

You can phone the Health Research Ethics Committee at (……) if there still is something that your study doctor has not explained to you, or if you have a complaint.

You will receive a copy of this information and consent form for you to keep safe.

### Declaration by participant

By signing below, I ………………………………….…………. agree to take part in a research study entitled Study of patient-reported pain during Bone Marrow Aspiration and Biopsy using local anaesthesia with intravenous midazolam co-administration at Tygerberg Hospital in South Africa.

I declare that:

- I have read this information and consent form, or it was read to me, and it is written in a language in which I am fluent and with which I am comfortable.
- I have had a chance to ask questions and I am satisfied that all my questions have been answered.
- I understand that taking part in this study is **voluntary,** and I have not been pressurised to take part.
- I may choose to leave the study at any time and nothing bad will come of it – I will not be penalised or prejudiced in any way.
- I may be asked to leave the study before it has finished, if the study doctor or researcher feels it is in my best interests, or if I do not follow the study plan that we have agreed on.

Signed at (*place*) ......................…........………… on (*date*) …………....………. 2019.

Signature of participant Signature of witness

### Declaration by investigator

I *(name)* …………………………………………….……… declare that:

- I explained the information in this document in a simple and clear manner to ………………………………….
- I encouraged him/her to ask questions and took enough time to answer them.
- I am satisfied that he/she completely understands all aspects of the research, as discussed above.
- I did/did not use an interpreter. (*If an interpreter is used then the interpreter must sign the declaration below.)*

Signed at (*place*) ......................…........…………… on (*date*) …………....……… 2019.

Signature of investigator Signature of witness
