# Supplementary material for: A study of patient‐reported pain during bone marrow aspiration and biopsy using local anesthesia alone compared with local anesthesia with intravenous midazolam coadministration at a tertiary academic hospital in South Africa
Source: Health Sci Rep. 2022 Oct 31;5(6):e902. doi: 10.1002/hsr2.902 (PMC9621466; doi:10.1002/hsr2.902)
Supplement: Supplementary file 4 — Supporting information. [file HSR2-5-e902-s003.doc]

# INCWADANA YEENKCUKACHA ZOMTHATHI-NXAXHEBA NEFOMU YOKUNIKA IMVUME

| **Isihloko seProjekthi yoPhando:** | |
| --- | --- |
| Kuphononongo lwesigulane kwafumaneka ingxelo yokuva ubuhlungu xa kuTsalwa uMongo eThanjeni naxa kusenziwa uxilongo lomzimba (iBhiyopsi) ngokusebenzisa isithomalalisi-zintlungu kwimithambo-luvo sesiqhelo xa kufakwa iyeza le-midazolam emthanjeni kwiSibhedlele saseTygerberg eMzantsi Afrika. | |
| **IINKCUKACHA ZOMPHANDI OYINTLOKO (PI):** | |
| **Isihlonipho, igama lokuqala, ifani: UGqr. Fatima Alzanad** | **Inombolo yesalathiso sokuziphatha:**  S19/03/066 |
| **Idilesi yeposi ngokupheleleyo:** | **Inombolo yoqhagamshelwano yoMphandi oyiNtloko:** |

Singathanda ukukumema ukuba uthathe inxaxheba kwiprojekithi yophando. Nceda uthathe ixesha ufunda iinkcukacha ezibhalwe apha, eziza kucacisa ngeenkcukacha zale projekthi. Nceda ubuze kubasebenzi bophononongo okanye kugqirha wophononongo malunga nayo nayiphi na imibuzo onayo ngokuphathelele nayiphi na indawo ongayiqondi kakuhle kule projekthi. Kubaluleke kakhulu ukuba waneliseke ngokupheleleyo ukuba ukuqonda ngokucacileyo okuqulathwe kolu phando nendlela onokubandakanyeka ngayo. Kananjalo, ukuthatha kwakho inxaxheba **ukwenza ngokuzithandela ngokupheleleyo,** kwaye uvumelekile ukuba ungala inxaxheba. Ngenye indlela, ungazikhethela ukuthatha inxaxheba, okanye ungakhetha ukungathathi nxaxheba. Akukho nto imbi izakwenzeka ukuba uthi hayi: oku akusayi kukuchaphazela kakubi nangayiphi na indlela. Ukwala ukuthatha inxaxheba akusayi kuquka sohlwayo okanye ukulahlekelwa ngamalungelo okanye ukuncitshiswa komgangatho wokhathalelo oluselungelweni lakho. Ukhululekile ukuba ungarhoxa kolu phononongo nanini na, nokuba uqale ngokuvuma ukuthatha inxaxheba.

Olu phononongo luvunyiwe yiKomiti yokuziPhatha kuPhando lwezeMpilo yeYunivesithi yaseStellenbosch. Olu phononongo luza kuqhutywa ngokwezikhokelo zokuziphatha nemithetho-siseko yesiBhengezo saseHelsinki sehlabathi, iziKhokelo zaseMzantsi Afrika zoKwenziwa koMsebenzi weZonyango ngokuFanelekileyo (2006), iziKhokelo zoKuziphatha zeBhunga loPhando lwaMayeza kuPhando (2002), kunye neMithetho yokuZiphatha yeSebe lezeMpilo kuPhando lweZonyango: Imithetho-siseko, iiNkqubo naMaphononongo (2015).

## Ingaba lumalunga nantoni olu phononongo lophando?

Olu phononongo lwenzelwa ukufumanisa ukuba ingaba amayeza esiwasebenzisayo ngoku ekwenzeni uxilongo lomzimba (ibhayopsi) lokutsala umongo ethanjeni alungile ngokufanelekileyo kusini na ukuqinisekisa ukuba isigulane asivi buhlungu ngethuba kusenziwa inkqubo yoxilongo lomzimba (ibhiyopsi).

Ngoko ke siyakuthi sibuze imibuzo kuzo zonke izigulane esizenze uxilongo lomzimba (ibhiyopsi) lokutsala umongo ethanjeni ukuze sifumanise ukuba ingaba zithe zeva ubuhlungu kusini na ngethuba kusenziwa le nkqubo okanye hayi.

Kwiimeko apho zathi zeva nabuphi na ubuhlungu, sifuna ukwazi ukuba zafumana ubuhlungu kangakanani na.

Kuye kwenziwa inkqubo yoxilongo lomzimba (ibhayopsi) lokutsalwa komongo ethanjeni lakho kwaye sinomdla kakhulu wokwazi ngamava wakho.

## Kutheni sikucelile ukuba uthathe inxaxheba?

Ukhethiwe ukuba ubeyinxalenye yophononongo ngenxa yokuba ukhe wenziwa uxilongo lomzimba (ibhayopsi) lokutsalwa komongo ethanjeni lakho kwigumbi loqhaqho elikwibhuloko engu-X. Ngoko ke sifuna ukwazi ngamava wakho.

## Luya kuba yintoni uxanduva lwakho?

Uza kucelwa ukuba uphendule imibuzo ethile, neyakuthi ibekwakweli phepha lemibuzo lifanayo lazo zonke izigulane ezithatha inxaxheba kolu phononongo. Uxanduva lwakho kukuphendula yonke imibuzo ngokunyanisekileyo kangangoko unokukhumbula. Sicela imizuzu engagqithanga kwemi-5 ukuya kweli-10 yexesha lakho.

## Ingaba uya kuzuza ngokuthatha kwakho inxaxheba kolu phando?

Akukho nzuzo kuwe ubuqu. Kananjalo, olu phononongo luyakuthi lusincede ekuthatheni isigqibo seyona ndlela ifanelekileyo yokuqinisekisa ukuba izigulane ezenziwa uxilongo lomzimba (ibhayopsi) lokutsalwa komongo ethanjeni azinangxaki yokufumana nabuphi na ubuhlungu.

## Ingaba kukho nayiphi na imingcipheko ebandakanyekayo ngokuthatha kwakho inxaxheba kolu phando?

Olu phononongo alunamngcipheko umandundu kuwe. Kananjalo, ngenxa yokuba usenokuba namava wokufumana ubuhlungu, ukubanenkumbulo yobuhlungu obunjalo kubonakaliswe njengobubangela ixhala nokungonwabi kwizigulane ezithile. Ukuba ngexesha lemibuzo, uziva ukhathazekile okanye unexhala, nceda uchazele iqela lethu lophononongo kwaye wazi ukuba ukhululekile ukuba ungayeka ukubuzwa imibuzo. Ukuba uziva ukhathazekile okanye unexhala emveni kokubuzwa imibuzo, nceda wazise iqela lophononongo kwaye siyakuthi sikugqithisele kuMnu. Wood ofumene uqeqesho lokunceda wena.

## Ukuba akuvumi ukuthatha inxaxheba, zeziphi ezinye iindlela ezinokusetyenziswa onazo?

Ungazikhethela ngokwakho ukuba uyafuna na ukuthatha inxaxheba kolu phononongo okanye awufuni. Ukuba uyavuma ukuthatha inxaxheba kolu phononongo, ungarhoxa nangeliphi na ixesha ngaphandle kweziphumo ezingalunganga. Usenako ukwala ukuphendula nayiphi na imibuzo ongafuni ukuyiphendula ngaphandle kwengxaki yokufumana iziphumo ezingalunganga.

## Ngubani oza kubanako ukufikelela kwiinkcukacha zakho zonyango ezigciniweyo?

Ngu Gqr. Fatima Al Zanad kuphela (umphandi oyintloko) noGqr. Zivanai Chapanduka (isekela lakhe) abayakuthi bafikelele kwiinkcukacha ezigciniweyo.

Ifomu yokunika imvume esayiniweyo kunye namanye amaxwebhu aqulethe iinkcukacha ezichongekayo ziyakuthi zigcinwe kwindawo ekhuselekileyo netshixiweyo.

Nantoni na enokusetyenziswa ekuchongeni wena iyakuthi isuswe kwaye itshatyalaliswe ngoko nangoko kwaye ukufikelela kwiinkcukcha zophando ezigciniweyo kuyakuthi kusekelwe phezu kwemfuneko efanelekileyo yokubanolwazi lwesiseko sazo nangemvume yooGqirha abangu-Al Zanad noChapanduka.

Kuyakuthi kwenziwe isishwankathelo seziphumo kwaye kunikwe ingxelo ngokweqela lonke. Igama lakho kunye naziphi na ezinye iinkcukacha ezinokuthi zichonge wena akusayi kwabelwana ngazo. Xa kupapashwa okufunyanisiweyo kolu phononongo okanye kusenziwa umboniso wako kwiinkomfa zenzululwazi siyakuthi siqinisekise ukuba awuchazwa ukuba ungubani.

Ingaba uza kuhlawulwa ngokuthatha inxaxheba kolu phononongo kwaye ingaba kukho naziphi na iindleko ezibandakanyekayo?

Awusayi kufumana ntlawulo ngokuphendula imibuzo.

Ingaba ikhona na enye into ekufuneka ukuba uyazi okanye uyenze?

Nceda ufowunele uGqr. Fatima Alzanad ngokukhululekileyo ku- (......) okanye uGqr. Chapanduka ku-(.........) ukuba unayo nayiphi na eminye imibuzo onayo okanye ufumana naziphi na iingxaki.

Ungafowunela iKomiti yokuZiphatha kuPhando lweZempilo ku-(.........) ukuba kusekho into athe ugqirha wakho wophononngo akayicacisa kakuhle kuwe, okanye ukuba unesikhalazo.

Uza kufumana ikopi yezi nkcukacha nefomu yokunika imvume ukwenzela ukuba uzigcinele yona ngokukhuselekileyo.

### IsiBhengezo somthathi-nxaxheba

Ngokutyikitya apha ngezantsi, mna …………………………………..…………. ndiyavuma ukuthatha inxaxheba kuphononongo lophando olunesihloko esithi Kuphononongo lwesigulane kwafumaneka ingxelo yokuva ubuhlungu xa kuTsalwa uMongo eThanjeni naxa kusenziwa uxilongo lomzimba (iBhiyopsi) ngokusebenzisa isithomalalisi-zintlungu kwimithambo-luvo sesiqhelo xa kufakwa iyeza le-midazolam emthanjeni kwiSibhedlele saseTygerberg eMzantsi Afrika.

Ndibhengeza okokuba:

- Ndizifundile ezi nkcukacha kunye nefomu yokunika imvume, okanye ndiyifundelwe, kwaye ibhalwe ngolwimi endilwaziyo nendiziva ndikhululekile ngalo;
- Ndiye ndaba nethuba lokubuza imibuzo kwaye ndanelisekile kuba yonke imibuzo yam iphenduliwe.
- Ndiyaqonda ukuba ukuthatha kwam inxaxheba kolu phononongo ndikwenza **ngokuzithandela,** kwaye khange ndinyanzelwe ukuba ndithathe inxaxheba.
- Ndingakhetha ukuyeka kuphononongo nangeliphi na ixesha kwaye akukho nto imbi iyakuthi yenzeke ngenxa yoko – andiyi kufumana sohlwayo okanye ndicalulwe nangayiphi na indlela.
- Ndisenokucelwa ukuba ndilushiye uphononongo lungekapheli, ukuba ugqirha wophononongo okanye umphandi ucinga ukuba oko kundifanele ngcono, okanye ukuba isicwangciso sophononongo andisilandeli ngale ndlela sivumelene ngayo.

Sityikityelwe (*indawo*) e......................…........…………….. ngomhla (*umhla*) we- …………....……….. 2019.

Utyikityo lomthathi-nxaxheba Utyikityo lwengqina

### Isibhengezo somphandi

Mna (*igama*) ……………………………………………..……… ndazisa okokuba:

- Ndimcacisele u .....................................................ngeenkcukacha ezikolu xwebhu ngendlela elula nangokucacileyo
- Ndimkhuthazile ukuba abuze imibuzo kwaye athathe ixesha elaneleyo ukuba ayiphendule.
- Ndanelisekile kukuba uyiqonda ngokwaneleyo yonke imiba yolu phando, njengoko icacisiwe apha ngentla.
- Ndiyisebenzisile/andiyisebenzisanga itoliki. (*Ukuba kusetyenziswe itoliki, loo toliki mayityikitye esi sibhengezo silapha ngezantsi).*

Sityikityelwe (*indawo*) e......................…........…………….. ngomhla (*umhla*) we- …………....……….. 2019.

Utyikityo lomphandi Utyikityo lwengqina
