# Supplementary material for: A study of patient‐reported pain during bone marrow aspiration and biopsy using local anesthesia alone compared with local anesthesia with intravenous midazolam coadministration at a tertiary academic hospital in South Africa
Source: Health Sci Rep. 2022 Oct 31;5(6):e902. doi: 10.1002/hsr2.902 (PMC9621466; doi:10.1002/hsr2.902)
Supplement: Supplementary file 5 — Supporting information. [file HSR2-5-e902-s002.docx]

INLIGTINGSBLAD EN TOESTEMMINGSVORM VIR DEELNEMERS

| **Titel van die navorsingsprojek:** | |
| --- | --- |
| Studie van pasiënt-aangemelde pyn tydens beenmurgaspirasie en ‑biopsie met gebruik van plaaslike verdowing met gelyktydige toediening van intraveneuse midasolaam by Tygerberg Hospitaal in Suid-Afrika. | |
| **INLIGTING OOR HOOFNAVORSER (HN):** | |
| **Titel, voornaam, van: Dr Fatima Alzanad** | **Etiek-verwysingsnommer:**  S19/03/066 |
| **Volledige posadres:** | **HN se kontaknommer:** |

Ons wil u graag nooi om aan ’n navorsingsprojek deel te neem. Lees asseblief rustig deur die inligting op hierdie blaadjie, want dit verduidelik mooi hoe die projek werk. Vra gerus die studiepersoneel of ‑dokter as daar enige deel van hierdie projek is wat u nie heeltemal verstaan nie. Dit is baie belangrik dat u heeltemal tevrede moet wees dat u deeglik verstaan waaroor die navorsing gaan en hoe u daarby betrokke kan wees. Onthou ook, u deelname is **heeltemal vrywillig**, en u kan ook weier om deel te neem. Met ander woorde, u kan kies om deel te neem, of om nie deel te neem nie. Niks slegs sal gebeur as u besluit om nee te sê nie: dit sal glad nie enige slegte gevolge vir u inhou nie. As u weier om deel te neem, sal u nie gestraf word of enige voordele verloor nie, en die vlak van sorg waarop u andersins geregtig is, sal ook nie verlaag word nie. U kan ook in enige stadium aan die studie onttrek, selfs al het u aan die begin ingestem om deel te neem.

Hierdie studie is deur die Universiteit Stellenbosch se Gesondheidsnavorsingsetiekkomitee goedgekeur. Die studie sal uitgevoer word volgens die etiekriglyne en -beginsels van die internasionale Helsinki-verklaring, die Suid-Afrikaanse riglyne vir goeie kliniese praktyk (2006) en die Mediese Navorsingsraad (MNR) se navorsingsetiekriglyne (2002), en die Departement van Gesondheid se Ethics in Health Research: Principles, Processes and Studies (2015).

## Waaroor gaan hierdie navorsingsprojek?

Hierdie studie wil uitvind of die medisynes wat ons tans gebruik om beenmurgbiopsies te doen, goed genoeg is om te verseker dat die pasiënt nie tydens die biopsieprosedure pyn ervaar nie.

Ons sal dus al die pasiënte op wie ons beenmurgbiopsies gedoen het, vrae vra om uit te vind of hulle tydens die prosedure pyn ervaar het of nie. In gevalle waar daar wel pyn ervaar is, wil ons uitvind hoeveel pyn hulle ervaar het.

U het ’n beenmurgbiopsieprosedure ondergaan, en ons stel dus baie belang om uit te vind wat u ervaring was.

## Hoekom nooi ons u om deel te neem?

U word gekies om aan die studie deel te neem omdat ’n beenmurgbiopsie in die X-blokteater op u uitgevoer is. Ons wil dus graag meer weet oor u ervarings.

## Wat sal ons van u verwag?

U sal gevra word om ’n paar vrae te beantwoord, en dieselfde vrae sal gevra word aan alle pasiënte wat aan hierdie studie deelneem. U verantwoordelikheid is om al die vrae eerlik te beantwoord so ver as wat u kan onthou. Ons vra u nie vir meer as 5–10 minute van u tyd nie.

## Watter voordeel is daar vir u as u aan hierdie navorsing deelneem?

Daar is geen direkte voordele vir u as individu nie. Die studie sal ons egter help om te besluit wat die beste manier is om seker te maak dat pasiënte wat beenmurgbiopsies ondergaan, nie enige pyn ervaar nie.

## Hou u deelname aan hierdie navorsing enige risiko’s in?

Hierdie studie hou geen ernstige risiko’s vir u in nie. Daar is egter al getoon dat, as gevolg van die moontlikheid dat hulle dalk pyn ervaar het, sommige pasiënte angs en ongelukkigheid beleef as hulle daardie pyn onthou. As u omgekrap of angstig voel terwyl u die vrae beantwoord, sê dit asseblief vir die navorsingspan, en onthou dat dit u vry staan om die ondervraging te staak. As u ná die ondervraging omgekrap of angstig voel, sê dit asseblief vir die navorsingspan en ons sal u verwys na mnr. Wood, wat opgelei is om u by te staan.

## Watter alternatiewe is daar as u besluit om nié deel te neem nie?

U kan kies of u aan hierdie studie wil deelneem of nie. Selfs al stem u in om aan hierdie studie deel te neem, kan u in enige stadium onttrek sonder dat dit enige negatiewe gevolge vir u sal inhou. U kan ook weier om enige vrae te beantwoord wat u nie wil nie, sonder dat dit enige negatiewe gevolge vir u sal inhou.

## Wie sal kan kyk wat in u mediese lêers staan?

Slegs dr Fatima Al Zanad (die hoofnavorser) en dr Zivanai Chapanduka (haar studieleier) sal toegang tot die inligting hê.

Die ondertekende toestemmingsvorm en ander dokumente wat data bevat wat u kan identifiseer, sal op ’n veilige plek toegesluit word.

Enige item wat gebruik kan word om u te identifiseer, sal so gou as moontlik verwyder en vernietig word en toegang tot navorsingsdata sal slegs gegee word aan persone wat dit moet hê, en dan slegs met drr. Al Zanad en Chapanduka se toestemming.

Die resultate sal opgesom en gesamentlik in ’n verslag gebruik word. U naam of enige ander inligting wat u kan identifiseer, sal nie met enigiemand gedeel word nie. Wanneer die bevindings van hierdie studie gepubliseer of by wetenskaplike konferensies aangebied word, sal ons verseker dat u identiteit nie bekend word nie.

Sal u betaal word om aan hierdie proefneming deel te neem, of sal dit u enigiets kos?

U sal nie betaal word om die vrae te beantwoord nie.

Is daar enigiets anders wat u moet weet of doen?

U is welkom om dr Fatima Alzanad by (......) of dr Chapanduka by (............) te skakel indien u enige verdere vrae het of enige probleme ondervind.

U kan ook die Gesondheidsnavorsingsetiekkomitee by 021 938 9677/9819 skakel indien daar steeds iets is wat u studiedokter nie aan u verduidelik het nie, of indien u ’n klagte het.

U sal ŉ afskrif van hierdie inligtingsblad en toestemmingsvorm kry om saam te neem huis toe en veilig te bewaar.

### Verklaring deur deelnemer

Deur hieronder te teken, stem ek, …………………………………..…………., in om deel te neem aan ’n navorsingstudie getiteld Studie van pasiënt-aangemelde pyn tydens beenmurgaspirasie en ‑biopsie met gebruik van plaaslike verdowing met gelyktydige toediening van intraveneuse midasolaam by Tygerberg Hospitaal in Suid-Afrika.

Ek verklaar soos volg:

- Ek het hierdie inligtings- en toestemmingsvorm gelees, of dit is aan my voorgelees, en dit is geskryf in ’n taal wat ek vlot verstaan en waarmee ek gemaklik voel.
- Ek het geleentheid gehad om vrae te vra, en ek is tevrede dat al my vrae goed genoeg beantwoord is.
- Ek verstaan dat deelname aan hierdie studie **vrywillig** is en ek is nie onder druk geplaas om deel te neem nie.
- Ek besef ek kan in enige stadium ophou deelneem en niks slegs sal gebeur nie – ek sal ook geensins gestraf of benadeel word nie.
- Die navorsingspan kan my vra om my aan die studie te onttrek voordat dit afgehandel is as die studiedokter of navorser dink dit is in my beste belang, of as ek nie hou by die studieplan waarop ons ooreengekom het nie.

Geteken te (*plek*) ......................…........…………….. op (*datum*) …………....……….. 2019.

Handtekening van deelnemer Handtekening van getuie

### Verklaring deur navorser

Ek, (*naam*) ……………………………………………..………, verklaar soos volg:

- Ek het die inligting in hierdie dokument op ’n eenvoudige en duidelike manier aan…………………………………..verduidelik.
- Ek het hom/haar aangemoedig om vrae te stel en het genoeg tyd daaraan afgestaan om die vrae te beantwoord.
- Ek is tevrede dat hy/sy alle aspekte van hierdie navorsing, soos dit hierbo uiteengesit is, ten volle verstaan.
- Ek het (nie) ŉ tolk gebruik (nie). *(Indien 'n tolk gebruik is, moet die tolk die verklaring hieronder teken.)*

Geteken te (*plek*) ......................…........…………….. op (*datum*) …………....……….. 2019.

Handtekening van ondersoeker Handtekening van getuie
